# Mael is essential for cancer cell survival and tumorigenesis through protection of genetic integrity

**DOI:** 10.18632/oncotarget.13756

**Published:** 2016-12-01

**Authors:** Su-Hyeon Kim, Eun-Ran Park, Eugene Cho, Won-Hee Jung, Ju-Yeon Jeon, Hyun-Yoo Joo, Kee-Ho Lee, Hyun-Jin Shin

**Affiliations:** ^1^ Division of Radiation Cancer Research, Korea Institute of Radiological & Medical Sciences, Seoul 139-706, Republic of Korea

**Keywords:** Mael, oncogenic transformation, genetic integrity, oncogene, ATM

## Abstract

Germ line-specific genes are activated in somatic cells during tumorigenesis, and are accordingly referred to as cancer germline genes. Such genes that act on piRNA (Piwi-interacting RNA) processing play an important role in the progression of cancer cells. Here, we show that the spermatogenic transposon silencer maelstrom (Mael), a piRNA-processing factor, is required for malignant transformation and survival of cancer cells. A specific Mael isoform was distinctively overexpressed in diverse human cancer cell lines and its depletion resulted in cancer-specific cell death, characterized by apoptosis and senescence, accompanied by an increase in reactive oxygen-species and DNA damage. These biochemical changes and death phenotypes induced by Mael depletion were dependent on ATM. Interestingly Mael was essential for Myc/Ras-induced transformation, and its overexpression inhibited Ras-induced senescence. In addition, Mael repressed retrotransposon activity in cancer cells. These results suggest that Mael depletion induces ATM-dependent DNA damage, consequently leading to cell death specifically in cancer cells. Moreover, Mael possesses oncogenic potential that can protect against genetic instability.

## INTRODUCTION

Members of the Piwi protein family, which form ribonucleoprotein complexes that process Piwi-associated RNAs (piRNAs), function to silence retrotransposons and other repeat elements during spermatogenesis by degrading transcripts or regulating epigenetic modulation of chromatin, thereby maintaining genetic stability [1–3]. It was recently reported that piRNAs are expressed across somatic tissue; relatively small number of known piRNAs are expressed [4] and function in transposon silencing and epigenetic programming in somatic tissue [5]. Some Piwi subfamily members are overexpressed in various types of cancers and have been suggested to play a role during tumorigenesis. Inactivation of the several piRNA-processing factors suppresses malignant growth in Drosophila [6], and orthologs of some of these genes that plays a role in tumor development, are overexpressed in a variety of human somatic tumors [6–9].

The spermatogenic transposon silencer Mael (maelstrom) is a PIWI-interacting protein that localizes to both perinuclear nuage and nuclei [10–13]. Testis germ cells in Mael knockout mice show a defect in spermatogenesis as a result of uncontrolled expression of transposable elements, DNA damage, and meiotic failure [12]. It has been speculated that Mael may function at steps downstream of piRNA biogenesis, such as shuttling Piwi complexes from nuage to the nucleus [12, 14].

Although Mael is predominantly expressed and functions in testis tissue, several reports have suggested that Mael is involved in cancer. Mael is overexpressed various cell lines derived from a majority of cancer types including lung, breast, prostate and colon through epigenetic modifications [15] and is overexpressed in epithelial ovarian cancer tissue [16] and promotes hepatocellular carcinoma metastasis [17]. Proteomic analysis reveals that Mael interacts with stress granule proteins in cancer cells [18].

Here, we investigated the roles of Mael in human somatic cancer cells. We found that Mael isoform 3 is overexpressed in tumor cells and HCC tissues, and Mael depletion increases DNA damage and reactive oxygen species (ROS) production, followed by apoptosis or senescence. We further found that the ATM is essential for Mael depletion-induced cytotoxicity. In addition, modulating Mael expression altered transformation of mouse embryo fibroblasts (MEFs) induced by oncogenic Myc or Ras, and Mael overexpression abolished Ras-induced senescence. Thus, we demonstrate a novel function of Mael in cancer cell survival and transformation through protection of genetic integrity, establish Mael as a potentially novel target in cancer therapy.

## RESULTS

### Mael is essential for survival of cancer cells, but not normal cells

**Figure 1:**
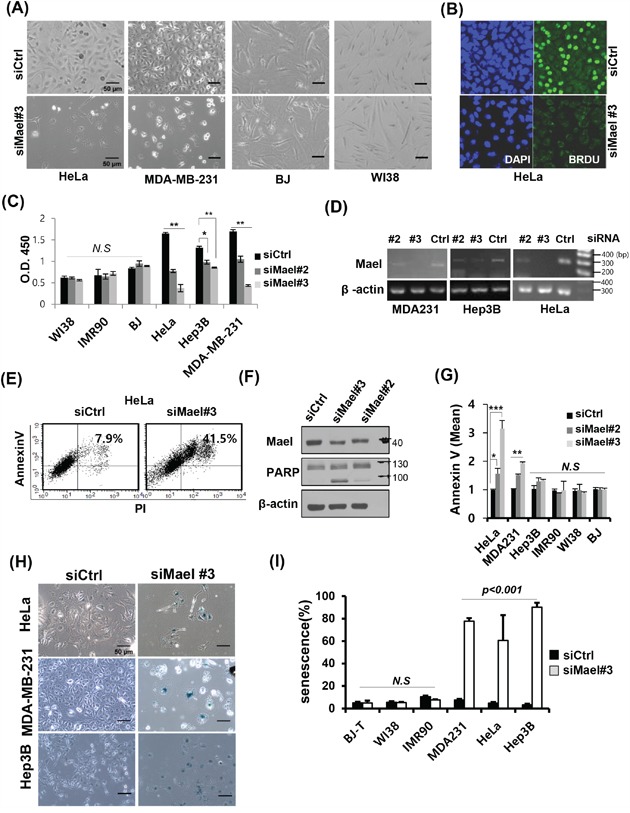
Mael depletion induces cancer cell-specific apoptosis and senescence **A**. Images of various Mael-depleted cancer cells and normal cells captured over 72 hours by light microscopy (100× magnification). **B**. The effect of Mael depletion on cell proliferation was analyzed by BrdU incorporation. Representative confocal images of Mael-depleted HeLa cells stained with BrdU (green) and DAPI (blue). **C**. The cell proliferation assay was performed using BrdU-based ELISA. siRNA-transfected cancer cells and normal cells were labeled with BrdU for 4 hours, and ELISA was performed according to the manufacturer's protocol. Statistical significance was determined by Student's *t*-test. Statistical differences are indicated (*p* < 0.01; **p < 0.001; *N.S*: non-significant). **D**. RT-PCR confirming siRNA-mediated knockdown. **E**. Apoptosis caused by Mael depletion in HeLa cells was determined by measuring annexin V/PI staining by flow cytometry and **F**. PARP cleavage by western blot analysis. **G**. Annexin V-positive cells from three individual experiments are depicted graphically. Statistical differences are indicated (*p < 0.05; **p < 0.01, ***p < 0.001). **H**. Representative microscopy images showing increased staining for the senescence marker β-galactosidase in Mael-depleted cancer cells. **I**. The summary data quantifying the results in H. This experiment was repeated three times and similar results were obtained.

To explore the role of Mael in tumor cells, we examined the phenotype of tumor cells following siRNA-mediated depletion of Mael. Upon transfection of Mael-siRNA, HeLa and MDA-MB-231 cancer cells exhibited a change in their shape towards an enlarged and flattened morphology, accompanied by massive detachment from the substrate (Figure 1A). In contrast to cancer cells, BJ and WI38 normal cells showed no such morphological change. Next, we examined the effect of Mael depletion on cell proliferation. BrdU (5′-bromo-2-deoxyuridine) incorporation was remarkably reduced in Mael-depleted HeLa (Figure 1B). To quantify this effect in various cells, a BrdU incorporation assay was also performed using a ELISA kit. Proliferation was significantly reduced by Mael depletion in all tested cancer cells (HeLa, Hep3B, and MDA-MB-231), while there was no difference in normal cells (BJ, WI-38, and IMR90) (Figure 1C). The RNA reduction by each siRNA targeting Mael was confirmed by both RT-PCR (Figure 1D) and real-time PCR (Supplementary Figure S1C). Further, colonogenic survival was severely reduced by Mael depletion in all tested cancer cell lines (Supplementary Figure S1A and S1B).

To determine whether the inhibition of cancer cell growth by Mael depletion is associated with cell death, we examined apoptosis using annexin V/PI staining. Mael depletion in HeLa cells significantly increased the annexin V/PI double-positive population (Figure 1E). Apoptosis induced by Mael depletion also confirmed at other cancer cell lines (Figure 1G, Supplementary Figure S1D). Consistent with this, PARP cleavage was detected in Mael-depleted HeLa cells (Figure 1F). To determine whether Mael depletion-induced inhibition of survival is also associated with senescence, we stained for the senescence marker β-galactosidase, in HeLa, MDA-MB-231, and Hep3B cells. Under conditions of Mael depletion, these cancer cell lines were positive for β-galactosidase staining (Figure 1H), and a quantitative analysis showed a substantial increase in the stained population (Figure 1I). Notably, β-gal–positive Hep3B cells were negative for annexin V staining (Figure 1G), despite showing severe inhibition of clonal survival (Supplementary Figure S1A, 1B) and proliferation (Figure 1C). These findings indicate that Mael depletion causes cancer cells to undergo cell death with apoptosis and/or senescence.

The effect of Mael on the survival of cancer cells was also confirmed with shRNAs targeting Mael. Both transfection of shRNA-encoding plasmids (Supplementary Figure S1E, S1G) and infection of shRNA-encoding lentivirus (Supplementary Figure S1F) resulted in reduced cell survival in the HeLa and MDA-MB-231cancer cell lines.

### Mael isoform 3 is overexpressed in diverse cancer cell lines

**Figure 2:**
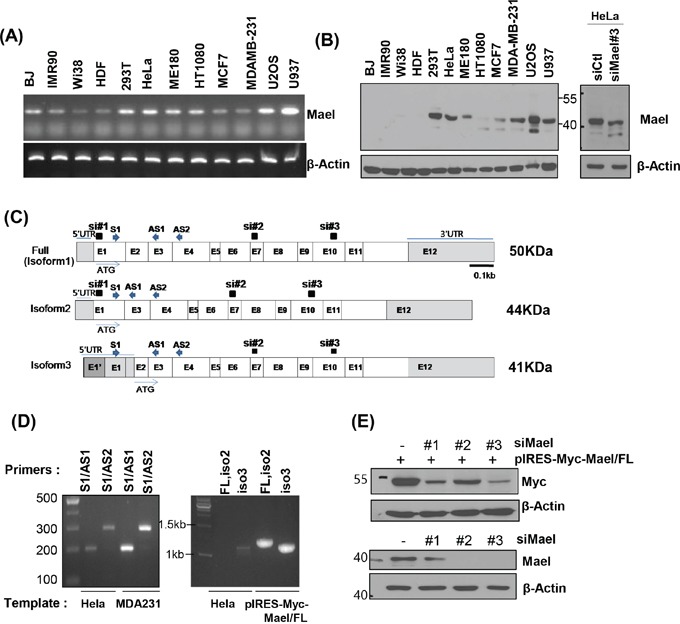
Mael isoform 3 is overexpressed in cancer cells **A, B**. Mael mRNA and protein expression in cells of various cancers and normal cells. The major protein band detected with the anti-Mael antibody at ~40 kD in HeLa cell lysate was smaller after Mael depletion. **C**. Schematic diagram of the three reported Mael isoforms, siRNA and primers are also depicted. **D**. RT-PCR performed using cDNA from HeLa and MDA-MB-231 cells with primers that yield different-sized amplicons for each isoform (left panel) and primers that amplify total coding sequences (full, Iso1, Iso2) (right panel). **E**. RT-PCR confirming the knockdown efficacy of three different siRNAs towards exogenous Mael isoform 1 (Iso1; upper blot) and endogenous Mael (lower blot) in HeLa cells.

Although Mael protein is barely detectable in most normal somatic tissues except testis, recent reports have shown that the protein is highly expressed in somatic cancer patient tissues and cancer cell lines [15–18]. Consistent with these reports, RT-PCR and Western blotting demonstrated Mael overexpression in tumor tissues of HCC patients compared with corresponding adjacent liver tissues (Supplementary Figure S2B). And we comprehensively examined Mael expression in a larger number of human cancer cell lines and normal cells. Mael transcript levels were higher in cancer cell lines than in normal cells (Figure 2A, Supplementary Figure S2A). Unexpectedly, we detected a Mael antibody-reactive protein smaller than the predicted molecular weight of Mael (50 kD) in diverse human cancer cell lines and HCC tissues (Figure 2B and Supplementary Figure S2B). siRNA-mediated Mael depletion decreased the level of this smaller protein in HeLa cells, confirming its identity as a bona fide Mael isoform.

Mael protein isoform 1 (~50 kD) which expresses at testis tissues as well as isoform 2 (~44 kD) and 3 (~41 kD) are recorded in National Center for Biotechnology Information (NCBI) database (Figure 2C). Isoform 2 lacks exon 2 owing to alternative splicing, and isoform 3 utilizes start codon in exon 3. To determine which isoform is expressed in cancer cell lines, we designed primers spanning exons 1–3 (S1/AS1) and 1–4 (S1/AS2) can distinguish isoform 2 from isoforms 1 and 3. We found that primer sets S1/AS1 and S1/AS2 generated PCR products 292 and 199 bp in size, respectively, rather than the 199 and 106 bp sizes expected for isoform 2 (Figure 2D, left panel). In addition, when we amplified three isoforms using primer sets that encompass the entire coding sequence, only isoform 3 was detected in HeLa cell-derived cDNA (Figure 2D, right panel), although both primer sets successfully amplified Mael isoforms from the plasmid (pIRES-Myc-Mael/FL). It also confirmed with siRNAs specifically targeting isoforms (siRNA#1: Iso1/2, siRNA #2, 3: Iso 1/2/3). Even though three siRNAs effectively reduced exogenous Mael expression, siRNA#1 didn't deplete endogenous protein (Figure 1E), further supporting that Mael is expressed as isoform 3 in cancer cells.

### Mael depletion induces spontaneous DNA damage and ROS specifically in cancer cells

**Figure 3:**
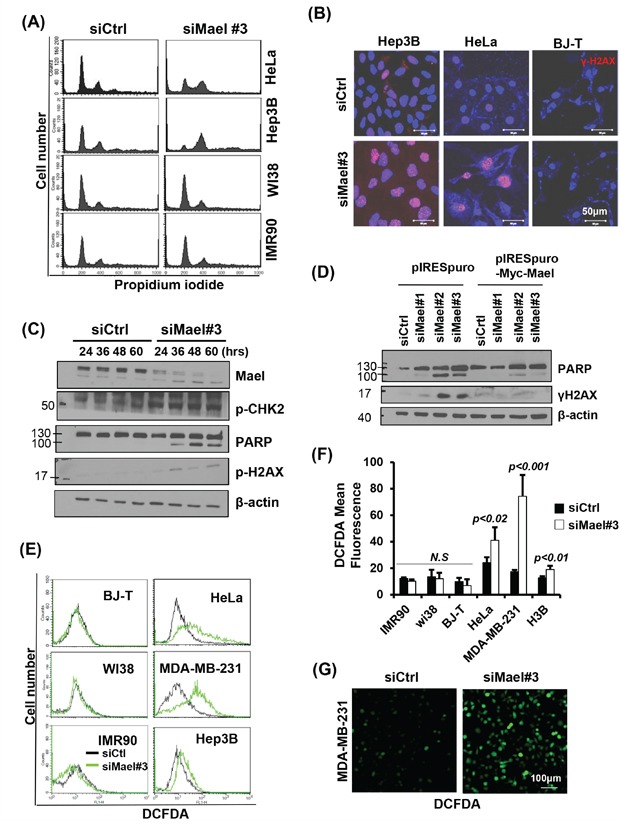
Mael depletion induces spontaneous DNA damage and ROS production in cancer cell lines **A**. Cell-cycle progression, monitored 72 hours after transfection, is depicted as a histogram of DNA content. **B, C**. The effects of Mael depletion on DNA damage were analyzed by immunofluorescence using an anti-γH2AX antibody and by western blotting using DNA damage-related antibodies. The protein lysates from HeLa cells were used for western blot analysis. **D**. Western blot detection of PARP cleavage in HeLa cells transfected with siRNAs selectively targeting specific Mael isoforms. **E**. Intracellular ROS levels in Mael-depleted cancer cells and normal cells, determined by measuring DCFDA fluorescence, were analyzed by flow cytometry and confocal microscopy. The FACS histogram of DCFDA intensity is a representative of three experiments. **F**. ROS levels in each experiment were quantified by measuring DCFDA mean fluorescence. The result was acquired from the average of three independent measurements. Statistical significance was determined by Student's *t*-test. **G**. intracellular ROS levels were visualized by microscopy.

To determine the underlying mechanistic basis of the cancer cell-specific death induced by Mael depletion, we first compared changes in the cell-cycle profile following Mael depletion between cancer and normal cell lines. Upon transfection of Mael-siRNA, HeLa and Hep3B cells exhibited enrichment in the G2/M phase of the cell cycle (Figure 3A). G2/M arrest occurs under various stress conditions, including DNA damage. Therefore, we next evaluated DNA damage using γ-H2AX as a DNA damage marker following Mael depletion. As shown in Figure 3B, γ-H2AX signals were increased in Mael-depleted HeLa and Hep3B cells, accompanied by an increase in nucleus size. Immunoblot analyses confirmed that Mael depletion caused DNA damage in HeLa cells, revealing an increase in PARP cleavage, phosphorylated Chk2 (pChk2) levels, and γ-H2AX expression (Figure 3C). Mael-siRNA #2 and #3 induced PARP cleavage and increased γ-H2AX, whereas Mael-siRNA#1 failed to do so (Figure 3D), confirming that the apoptotic cell death and associated DNA damage caused by Mael depletion reflects knockdown of isoform 3.

Next, we analyzed intracellular ROS level, since genotoxic stress is generally involved in the generation of ROS [19, 20]. Upon Mael-siRNA transfection, all three cancer cell lines showed a significant increase in DCFDA fluorescence, whereas no increase in DCFDA signal was observed in normal cells (Figure 3E, 3F), indicating that Mael depletion causes ROS generation specifically in cancer cells. These findings indicate that Mael depletion induces DNA damage in cancer cells, accompanied by generation of intracellular ROS. Fluorescence microscopy also revealed cells with a bright DCFDA signal after transfection of Mael-siRNA in MDA-MB-231 cells (Figure 3G). Thus, Mael in cancer cells appears to protect against DNA damage and associated ROS generation.

### Mael depletion-induced cytotoxicity is dependent on ATM

**Figure 4:**
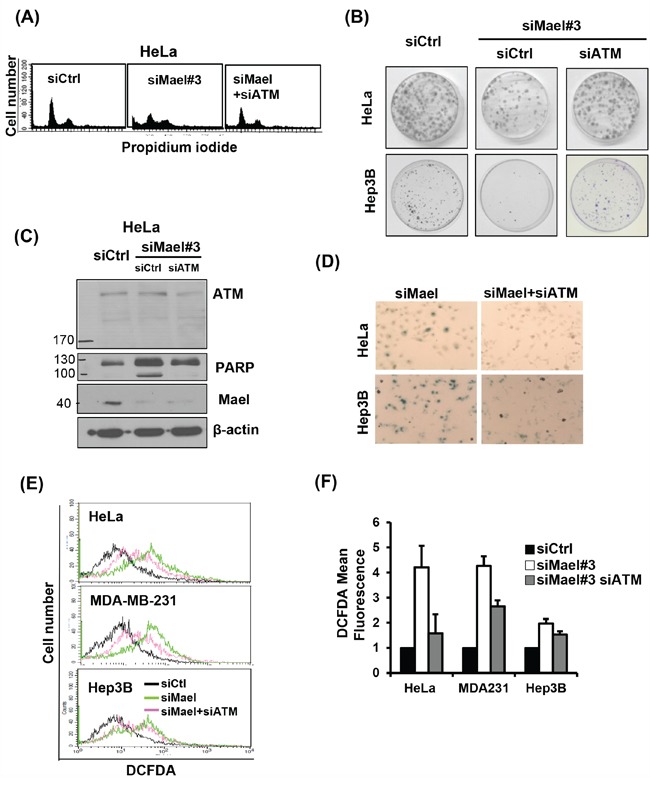
Mael depletion–induced cytotoxicity is dependent on ATM All the cytotoxic effects, including effects on **A**. the cell cycle; **B**. clonogenic cell survival; **C**. PARP cleavage; **D**. senescence; **E, F**. and ROS production; of Mael-targeted siRNA in cancer cell lines were reversed by ATM depletion.

To further elucidate the molecular basis of DNA damage-related changes induced by Mael depletion, we sought to identify DNA damage-sensing molecules that might provide a mechanistic link. ATM is an upstream sensor that recognizes DNA-damage signals generated in response to genotoxic stress [21]. Based on this previous report and our present findings, we reasoned that Mael-expressing cancer cells could not survive Mael depletion owing to the resulting DNA-damage signal and subsequent activation of the cell death pathway. To test this, we simultaneously knocked down both ATM and Mael in cancer cells and monitored cell survival. Interestingly, siRNA-mediated knockdown of ATM in the context of Mael depletion rescued clonal survival in HeLa and Hep3B cells (Figure 4B), indicating that ATM is essential for cell death induced by Mael depletion. The disruption of the cell cycle accompanied by the G2/M phase enrichment caused by Mael knockdown also disappeared in cells co-depleted of ATM (Figure 4A). Moreover, ATM depletion attenuated the phenotypic changes induced by Mael depletion in cancer cells, decreasing PARP cleavage (Figure 4C), senescent cell numbers (Figure 4D), and ROS production (Figure 4E and 4F). This phenotypic conversion was observed in HeLa and Hep3B cells (Figure 4B and 4D), as well as MDA-MB-231 cells (Figure 4E and 4F). These results suggest that ATM is an essential sensor that detects the DNA-damage signal induced by Mael depletion and conveys it to the cell death program.

### Mael is essential for Myc/Ras-induced transformation and suppresses oncogenic Ras-induced senescence

**Figure 5:**
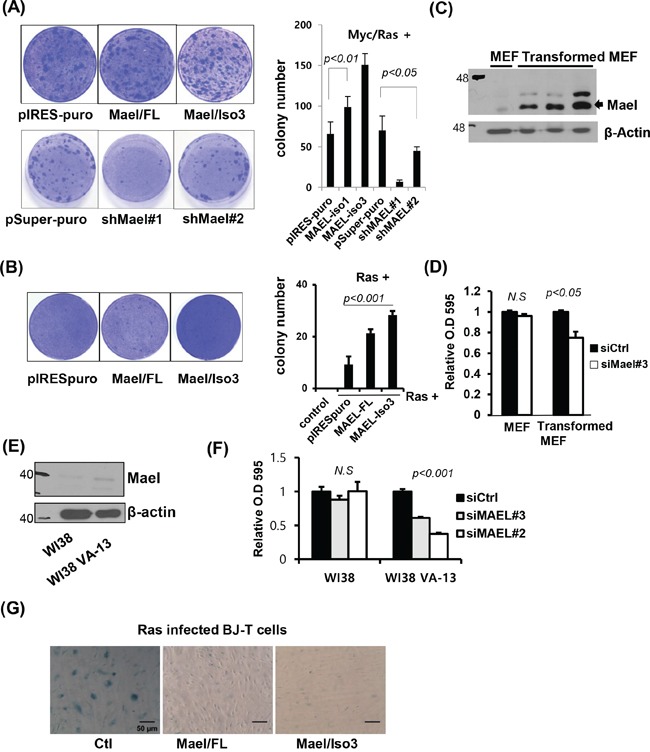
Mael is essential for Myc/Ras-induced transformation and suppression of oncogenic Ras-induced senescence **A, B**. *p53^−/−^* MEFs were co-transfected with Mael and Myc/Ras or Ras. Transformed colonies were visualized by crystal violet staining, and the colonies were counted in triplicates and the average number of colonies was calculated. **C**. MAEL protein expression was compared in MEF and Myc/Ras transformed MEF. **D**. Effects of mMael depletion on the viability of *p53^−/−^* MEFs and their transformed cells were tested using crystal violet staining. **E, F**. Mael protein expression and cellular responses to siRNA transfection were compared in non-transformed normal human WI38 cells and WI38 cells transformed with SV40Tag. **G**. β-galactosidase staining was performed to verify the effects of Mael on cellular senescence induced by infection with H-RasV12- expressing retrovirus in BJ-T cells.

The fact that Mael is overexpressed in tumor tissues and cancer cell lines, and is essential for cancer cell survival prompted us to investigate whether Mael has an impact on the generation of cancer. To explore this, we performed oncogene-induced transformed focus assays using *p53^−/−^* MEFs [22]. We cotransfected *p53^−/−^* MEFs with Mael isoform 3 or full-length Mael (isoform 1) together with Myc and Ras (Myc/Ras) or Ras alone. Both isoforms, but especially isoform 3, enhanced the efficiency of transformation induced by Myc/Ras, or Ras compared with cells transfected with pIRES-puro empty vector (Figure 5A, 5B). Conversely, depletion of Mael by cotransfection of either sh-Mael#1 or sh-Mael#2 decreased the number of transformed foci (Figure 5A). A comparison of MAEL expression level between untransformed and Myc/Ras-transformed *p53^−/−^* MEFs revealed that MAEL expression was increased during Myc/Ras transformation (Figure 5C). Consistent with the results obtained in human cancer cell lines, siRNA-mediated Mael depletion decreased the proliferation rate in transformed MEFs (Figure 5D). Combined with our observation that Mael is overexpressed in human cancer cells, these results suggest that Mael is an oncogene that increases tumorigenesis. To further assess this possibility, we examined Mael expression in a comparable set of normal human WI38 cells and their SV40 transformants, WI38 VA-13 cells. Similar to the results observed in MEFs, Mael expression was higher in transformed WI38 VA-13 cells than in parental WI38 cells (Figure 5E), indicating an increase in Mael expression during oncogenic transformation in human as well as mouse cells. Transfection of Mael-siRNA (#2 and #3) resulted in a significant decrease in the proliferation rate of WI38 VA-13 cells (Figure 5F). The results of these comparisons of human normal and transformed cells further support the conclusion that Mael isoform 3 has oncogenic potential to influence tumorigenesis.

The fact that Mael protects against DNA damage in cancer cell lines suggests that Mael could be associated with protection against DNA damage generated during transformation. To determine whether Mael influences oncogene-induced senescence, we stably expressed full-length Mael or isoform 3 in normal BJ-T cells and examined the effects of Mael expression on Ras-induced senescence. Retroviral infection of H-RasV12 resulted in an increase in the proliferation of BJ-T cells 5 days after infection. Approximately 10 days later, Ras-infected BJ-T cells showed a decrease in cell growth, a change in cell shape with a flattened and enlarged morphology, and an increase in β-galactosidase staining (Figure 5G). These senescence-associated phenotypic changes were remarkably attenuated in cells expressing Mael. Considering that Mael overexpression was detected during transformation, these results indicate that Mael acts by protecting against DNA damage and maintaining genomic integrity to play a role in allowing cells to escape Ras-induced senescence.

### Mael destailizes the mRNA of retrotransposons in cancer cell lines

**Figure 6:**
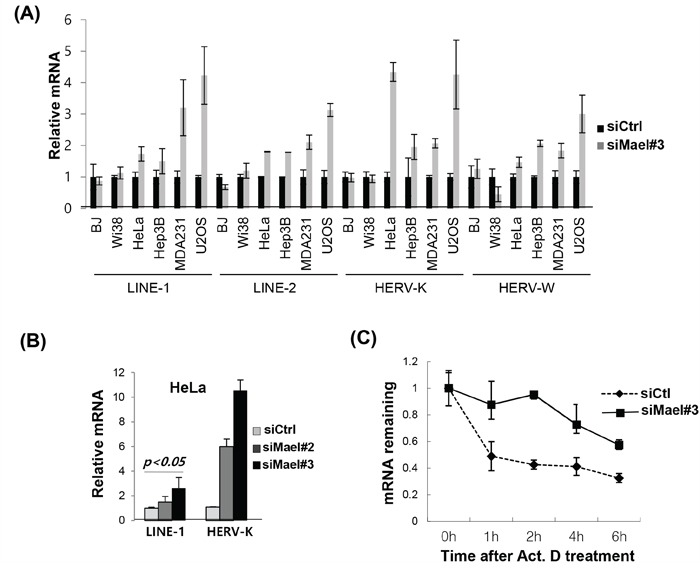
Mael depletion stabilizes the mRNA of retrotransposons in cancer cells **A, B**. Quantification of mRNA of the retrotransposons LINE-1, LINE-2, HERV-K, and HERV-W by real-time PCR using cDNA synthesized from Mael-depleted cancer cells and normal cells. C_t_ values for target mRNAs were normalized to those for GAPDH. Results were then calculated using the ΔΔC_t_ method, and are presented as mean ± SD. **C**. Evaluation of LINE-1 mRNA stability by real-time PCR. Transfected cells were treated with actinomycin D, and 18S rRNA was used as an internal control.

To determine whether Mael affects retrotransposon activity in somatic cancer cells, we measured the expression of the retrotransposable elements LINE-1 (ORF1), LINE-2, HERV-K (ORF1), and HERV-W in the cancer cell lines HeLa, MDA-MB-231, Hep3B, and U2OS and normal cells BJ, IMR90, and WI-38 following depletion of Mael. The mRNA of tested retrotransposons was clearly increased in cancer cell lines (Figure 3A). To accurately evaluate the activity of LINE-1 and HERV-K, we assayed for long terminal repeats (LTRs) and two different open reading frame (ORF) sequences present in each of these two transposable elements. We found that Mael depletion caused an increase in the expression of HERV-K and LINE-1 retrotransposons, LTR, ORF1 and ORF2 in all three cell lines examined, including HeLa, HT1080, and 293T (Supplementary Figure S3). Two different Mael-siRNAs showed similar increases in LINE-1 and HERV-K retrotransposon expression (Figure 6B). The degradation of retrotransposon mRNA during piRNA processing is essential for the maintenance of genomic integrity [12]. To determine whether Mael is involved in stabilizing retrotransposon mRNA, we analyzed the stability of LINE-1 mRNA after inhibition of transcription with actinomycin D. Within 1 hour after treatment with actinomycin D, LINE-1 mRNA levels were reduced in HeLa cells transfected with control-siRNA, indicating rapid degradation of LINE-1 mRNA in cancer cells (Figure 6C). In cells depleted of Mael by transfection of Mael-siRNA, LINE-1 mRNA levels remained relatively stable instead of rapidly decreasing. These findings suggest that Mael inhibits the activation of retrotransposons in cancer cells by promoting the degradation of mRNA for retrotransposable elements. Collectively, these data indicate that Mael, which is expressed abundantly in cancer cells, protects against DNA damage thereby maintaining genomic integrity.

## DISCUSSION

Genetic instability, which is affected by DNA repair processes and chromosome segregation, is a main cause and a common feature of cancer. Thus, the functions of many tumor-suppressor proteins affect cellular processes that fall into this category. On the other hand, cancer cells also develop machineries that facilitate their survival despite their increased genetic instability. Some of the mechanisms that serve these genetic-stabilizing functions are conserved between developmental and tumorigenic processes, reflecting the fact that cells in both contexts are highly proliferative. During development, genetic stability is finely tuned by various mechanisms, including Mael-related proteins. Many genes expressed during development are re-expressed in highly proliferating cancer cells, and many proto-oncogenes are involved in normal developmental growth and differentiation. We found that Mael maintains genetic stability in cancer cells; it is also crucial for transformation and inhibits Ras-induced senescence. We suggest that Mael serves to protect against cellular genotoxic stress in tumor cells and during the transformation process.

We showed that Mael depletion increases the mRNA stability of retrotransposons, suggesting that the function of Mael in tumors might be related to the piRNA pathway. In addition, we found that Mael isoform 3 is specifically expressed in cancer cell lines; this contrasts with germ tissues, where isoform 1 is expressed. Mael isoform 1 has an HMG domain, which is defective in isoform 3. This implies that the previously reported function of Mael as a chromatin remodeler or transcription factor is not related to its function in cancers. A recent study has reported that the HMG domain of Mael binds to and interacts with RNA hairpins rather than DNA [23]. RNA binding via the HMG domain provides structure-specific RNA binding. We found that isoform 3, which is predominantly expressed in various cancer cell lines, has a defective HMG domain and its function in transforming MEFs is more efficient. Further investigation will be required to discern the precise roles of isoform 3 and the HMG domain.

In the current report, we characterized the function of Mael protein in cancer cells, demonstrating that Mael depletion results in an increase in ROS, followed by apoptosis or senescence. We found that Mael is activated in proliferating cancer cells, where it serves to maintain genetic stability. It has been shown that Mael knockout mice are viable from embryos to adulthood, and matings of *Mael^+/−^* mice produce offspring in a normal Mendelian ratios [12], indicating that Mael function is not required in somatic cells or is mediated by other proteins. However, as we found here, expression of Mael is crucial for the survival of cancer cells. An additionally important property of Mael is its essential role in Myc/Ras-induced transformation, a role made more remarkable by the fact that Mael depletion in normal MEFs has little effect on cell survival. We thus speculate that Mael may contribute to the onset of cancer under physiological conditions. These observations have both therapeutic and biological implications. Because Mael protein is highly expressed in proliferating cancer cells as a survival factor, targeting it could be a useful therapeutic strategy.

## MATERIALS AND METHODS

### Cell lines and culture

The cell lines, HeLa, Hep3B, MDA-MB-231, ME-180, HT-1080, H1299, MCF7, U2OS, U937, Huh7, SK-HEP-1, WI38, WI38VA-13, IMR-90, BJ, and BJ-5ta (BJ-T), were purchased from ATCC, and SNU cell lines were purchased from Korean Cell Line Bank (KCLB). All the cells were cultured according to the repository's protocol. To maintain authenticity of the cell lines, frozen stocks were prepared at the second passage from the initial stocks, and every 3 months, a new frozen stock was used. *p53^−/−^* MEFs were prepared by crossing heterozygous *p53^+/−^* mice from the Jackson Laboratory. MEFs were maintained in Dulbecco's Modified Eagle Medium (DMEM) supplemented with 10% FBS and 1% antibiotics.

### Cell proliferation assay

To analyze cell proliferation, immunofluorescence- and ELISA-based BrdU incorporation assays were performed. For the immunofluorescence assay, HeLa cells were treated with siRNA for 48 hours and were labeled with 10 μM BrdU (Sigma-Aldrich, St. Louis, MO) for 4 hours, followed by treatment with 2 N HCl for DNA hydrolysis. Immunostaining was performed using anti-BrdU antibody (Abcam, Cambridge, UK, Ab1893). For ELISA, BrdU cell proliferation kit was used (Abcam, ab126556). The cells transfected with siRNAs for 24 hours in 60-mm dishes were reseeded into a 96-well plate at a density of 2 × 10^3^ and incubated for 36 hours. Then, cells were labeled with BrdU for 2 hours and the assay was performed according to the manufacturer's protocol. The absorbance was measured at 450 nm. All the samples were analyzed in triplicates.

For clonogenic assays, the test cell lines were plated in 60-mm dishes, transfected with siRNAs on the next day, and then incubated for 4–12 hours, depending on the cell line. Twenty-four hours after transfection, 5 × 10^2^ cells were seeded in 6-well plates; the assay was performed in triplicates. After 10–15 days of incubation, cells were fixed using 10% formaldehyde in phosphate-buffered saline (PBS) for 10 minutes, stained with crystal violet (0.1% w/v in 10% v/v ethanol) for 1 hour, and washed with water. The colonies of stained cells were counted.

For vital staining with crystal violet, cells were seeded at a density of 5 × 10^4^ cells/well in 6-well plates and then transfected the next day with siRNA. After 72 hours, the cells were fixed and stained using crystal violet. The washed cells were extracted with 1 mL 33% acetic acid, diluted 16-fold in acetic acid solution, and quantified by measuring absorbance at 595 nm.

### Western blot analysis

The proteins were extracted using lysis buffer, purified, separated by SDS-PAGE, and transferred onto nitrocellulose membranes. The following primary antibodies were used: anti-Mael (for human protein, Novus, NBP1-84359; for murine protein, Abcam #ab28661), anti-γ-H2AX (Ser139; Millipore, 05-636), anti-PARP (Cell Signaling, 9542), p-ATM (Ser1981; Cell Signaling, 2853), β-Actin (Santa Cruz Biotechnology, sc-47778), and pChk2 (T68; Abcam, Ab3501). Immunoreactive proteins were detected using enhanced chemiluminescence (ECL) reagents (Santa Cruz Biotechnology).

### Immunofluorescence staining

Cells were grown on coverslips in 6-well dishes and transfected. After 3 days, cells were fixed using ice-cold methanol and incubated for 5 minutes on ice. Cells were then dried for 5 minutes, rehydrated with PBS, and incubated with blocking solution (3% skimmed milk in PBS). Thereafter, cells were incubated with primary antibody against γ-H2AX (Upstate Biotechnology, Lake Placid, NY). After washing with PBS, cells were incubated with Alexa 488-conjugated secondary antibodies (Invitrogen, Carlsbad, CA), washed, and mounted in VECTASHIELD Mounting Medium containing 4′,6-diamidino-2-phenylindole (DAPI; H-1200).

### Senescence assay

Cells were fixed in 2% formaldehyde/0.2% glutaraldehyde, then washed with PBS (pH 6.0) and incubated at 37°C overnight with fresh SA-β-gal stain solution (1 mg/mL 5-bromo-4-chloro-indolyl-β-D-galactopyranoside; 1× PBS, pH 6.0; 2 mM MgCl_2_; 5 mM potassium ferricyanide; 5 mM potassium ferrocyanide). Blue-stained senescent cells were observed by standard light microscopy, and the percentage of SA-β-gal–positive cells was determined by counting 3 × 10^2^ cells in random fields on a slide.

### Flow cytometry

Cell cycle, apoptosis, and intracellular ROS were analyzed using flow cytometry. For cell cycle analysis, siRNA-transfected cells were harvested with trypsin at 60 hours post transfection, fixed with ethanol, incubated with RNase, and subsequently stained with 50 ng/mL propidium iodide (Sigma-Aldrich). The cell cycle profile was analyzed by flow cytometry using the FL2 channel (FACS Calibur, BD Biosciences). For apoptosis assays, cells were transfected with siRNA and incubated for 72 hours. Harvested cells were stained with FITC-conjugated annexin V antibody and PI, as per the manufacturer's protocol (BD Pharmingen), and were then analyzed by flow cytometry using FL1 and FL2 channels. ROS levels in cells transfected with siRNA for 48 hours were measured by incubating with DCFDA (10 μM) for 30 minutes. Cells were then harvested using trypsin, and intracellular ROS levels were analyzed in the FL1 channel using FACS Calibur.

### Transfection

Cells were transfected with a small inhibitor RNA (siRNA) duplex targeting Mael (Qiagen, Valencia, CA) at a final concentration of 20 nM using Lipofectamine RNA iMax (Invitrogen), according to the manufacturer's protocol. For studies examining combinatorial effects of other siRNAs, the total concentration of siRNAs was fixed at a final concentration of 20 nM. Detailed plasmid and siRNA sequence information is given in the supplementary materials and methods.

### RT-PCR and real time PCR

mRNA expression was analyzed by conventional reverse transcription PCR (RT-PCR) or quantified using real-time PCR. Total RNA was extracted using the Qiagen RNeasy Mini Kit (Qiagen) and reverse transcribed into cDNA using the iScript cDNA synthesis kit (Bio-Rad, Hercules, CA). The resulting cDNA was amplified using a Maxime PCR PreMix Kit (iNtRON Biotechnology, Korea) for conventional RT-PCR or using the SYBR green method (iQ SYBR Green supermix, Bio-Rad) for real-time quantitative PCR. Three replicates of real time PCR were performed for each analysis. Detailed primer information is given in the supplementary materials and methods.

### Transformed focus-forming assay

*p53^−/−^* MEFs were prepared by crossing heterozygous (*p53^+/−^*) mice, and were obtained from the Jackson Laboratory. Formation of transformed foci was assessed using early passage *p53^−/−^* MEFs cells (passage 2–4) co-transfected with 2 μg plasmids encoding *c-myc*, mutant H-RAS(V12), and test plasmids—pIRES-puro encoding hMael or pSuperpuro encoding sh-mMael. The cells were plated at a density of 6 × 10^5^ cells/100-mm dish and transfected 1 day later using the calcium phosphate precipitation technique. After 10–12 days of transfection, transformed foci were counted after staining with crystal violet.

## SUPPLEMENTARY MATERIALS FIGURES AND TABLES


