# Supplementary material for: Mael is essential for cancer cell survival and tumorigenesis through protection of genetic integrity
Source: Oncotarget. 2016 Dec 1;8(3):5026–37. doi: 10.18632/oncotarget.13756 (PMC5354889; doi:10.18632/oncotarget.13756)
Supplement: Supplementary file 1 [file oncotarget-08-5026-s001.pdf]

Mael is essential for cancer cell survival and tumorigenesis through protection of genetic integrity

SUPPLEMENTARY DATA

Plasmids

Human *MAEL* cDNA (isoform 1, FL) was amplified by polymerase chain reaction (PCR) with the gene-specific primer pair, 5′-ATGCCGAACCGTAAGGCCAGCCG-3′ and 5′-AGGGAAGTTGGGCCTGTTACT-3′, using cDNA isolated from the H358 cell line as a template. Amplified *MAEL* cDNA was cloned into the pIRES-puro3 and MFG-ires-puro vectors and tagged with Myc epitope at the N-terminus (pIRES-Myc-MAEL/FL). MAEL isoform 3 was amplified using pIRES-Myc-MAEL FL plasmid as a template and then cloned into the same vectors.

siRNA and shRNA

The primary Mael-targeting siRNA used was siMAEL#3 (5′-CATCAATAG TGTGACACCCAA-3′); other siRNAs (siMAEL#1, 5′-CCAGCCGGAATG CTTACTA-3′; siMAEL#2, 5′-TGAACGTGGGCATAA CCAA-3′) were used to confirm Mael-depletion effects or to identify specific Mael isoforms. The sequences of human Mael that target shRNAs for cloning into pLKO.1 were 5′-CAGCAATAGTGTGACACCCAA-3′ (#1, referred to siMael #3) and 5′-TGAACGTGGGCATAACCA A-3′ (#2). The siRNA sequence used to target human ATM was 5′-AATGGTGCTATTTACGGAGCT-3′. The sequence that targets shRNAs or siRNAs for murine Mael were 5′-GGAACTGGCCACCTATTTA-3′ (#1) and 5′-CAGCAACAG TGTGACACCCAA-3′ (#2).

Lenti-, retro-virus and Ras induced senescence assay

Ras induction of senescence was assessed by first preparing a Mael-, RAS-expressing retrovirus. 293T cells were cotransfected with MFG-puro, MFG-puro-MAEL/FL, MFG-puro-Mael/iso3, pBabe-puro or pBabe-puro-HRASV12 together with plasmids encoding the retroviral structural proteins, pGag-Pol and pVSV-G, using TurboFect (Thermo) as a transfection reagent. Supernatants containing recombinant retroviruses were harvested 24 and 48 hours after transfection and passed through a 0.45-µm syringe filter; polybrene was then added at a concentration of 8 µg/ml. BJ-T cells were infected with purified retroviruses. Three days after infection, infected cells were selected with puromycin (0.3 µug/ml). Stable cell lines were plated in 6-well plates and infected with retrovirus expressing H-RAS (V12). Ten days after infection, cells were analyzed for senescence.

The effect of Mael depletion on cancer cells was confirmed using shRNA-transduced lentivirus. The lentivirus was generated by transient transfection of lentiviral vector (pLKO.1-shMAEL #1, #2, and pLKO.1-backbone vector), together with psPAX2 (addgene) and pMD2.G (addgene) into 293T cells, and virus preparation and infection was conducted using the same protocol as used for retrovirus. At 48 h after infection, cancer cells were subdivided with 3×10^3 cells into 60-mm dishes in triplicate, and colonogenic cell survival was analyzed after 10 days.

Primers

Primer pairs for quantifying mRNA expression by RT-PCR or real-time PCR were used: Mael, 5′-TGGCCACTCTCTTTGGAATC-3′ (forward) and 5′-GCATTTCCAATT CTTCCAGC-3′ (reverse); and β-actin, 5′-ACCACACCTTCTACAATGAGC-3′ (forward) and 5′-CTTCATGATGGAGTTGAAGGT-3′ (reverse).

Specific isoforms (iso1, iso2, iso3) of Mael expressed in cancer cell lines were identified by RT-PCR using the primers S1 (5′-cgttgctgatgccatccctt-3′), AS1 (5′-ccaaag cagaatgtttcacctcc-3′) and AS2 (5′-ccatggcgagctacctcctc-3′), designed to amplify spliced transcript with different sizes, as follows: S1/AS1 (iso1, iso3: 199 bp; iso2: 106 bp) and S1/AS2 (iso1, iso3: 292 bp; iso2: 199 bp).

To quantify retrotransposon, residual genomic DNA from purified RNA was completely eliminated using a TURBO DNA-free kit (Ambion) and then cDNA was synthesized with the resulting DNA-free RNA using random hexamer primers. LINE-1, LINE-2, HERV-K and HERV-W were then quantified by real-time PCR using the following primer pairs: LINE-1-5′UTR, 5′-AAGGGGTGACGGTCGCACCTGGAA-3′ (forward) and 5′-AGCTGTGCTAGCAATCAGCGAGA-3′ (reverse); LINE-1-ORF1, 5′-TCCTCGAGA AGAGCAACTCCA-3′ (forward) and 5′-GGGTTTCTGCCGAGAGATCC-3′ (reverse); LINE-1-ORF2, 5′-ATGGCCATACTGCCCAA GGT-3′ (forward) and 5′-TGGCTTAGGATT GACTTG GCA-3′ (reverse); HERV-K-5′UTR, 5′-CAGATGCTTGAA GGCAGCAT-3′ (forward) and 5′-ACGTTGGACAA TACCTGGCT′3′ (reverse); HERV-K-ORF1, 5′-TGGG CAACCATTGTCGGGAAAC-3′ (forward) and 5′-GGCT

TATTCCCTGAAACACTTGGGA-3′ (reverse); and HERV-K-ORF2, 5′-GGCTTATTCCCTGAAACACTTGG GA-3′ (forward) and 5′-TCATCAAGGCTGCAA GCAGCATAC-3′ (reverse) ; LINE-2, 5′-CAGATCTC TCGG CAG AAACC-3′ (forward) and 5′-GCC TGGTGGTGACAAAATCT (reverse); HERV-W, 5′-GTTGTCCTG GAG GACTTG GA-3′ (forward) and 5′-TATGGGTACGGAGGGTTT CA-3′ (reverse) ; Glyceraldehyde-3-phosphate dehydrogenase (GAPDH), used as an internal control, was amplified with the primer pair, 5′-GAACATCATCCCTGCTCTTAC-3′ (forward) and 5′-CTCCGACGCCTGCTTCACC-3′ (reverse). For real-time PCR, mRNA expression was normalized to that of GAPDH, and relative expression was calculated using the $2^{-\Delta\Delta CT}$ method.

The mRNA stability of retrotransposons was measured by first treating transfected cells for 24 hours with 0.5 µg/ml actinomycin D to inhibit further transcription. Cells were then harvested 2, 4, and 6 hours after treatment and analyzed by real-time PCR as described above using 18S RNA as reference gene. The sequences of 18S primers were 5′-GGAGAG GGAG CCTGAGAAGCG-3′ and 5′-TTACAG GGCCTCGAA AGAGTC C-3′.

HCC specimens

Surgical specimens were obtained from patients undergoing treatment for HCC at a Korean cancer center hospital. A total of 30 samples were used for RT-PCR, and 15 samples were used for Western blotting. Tissues were surgically resected, and samples were immediately snap-frozen and stored in liquid nitrogen. Total RNA was isolated from frozen tissues using a Qiagen RNA isolation kit. This study was approved by the Institutional Review Boards of Korea Cancer Center Hospital.

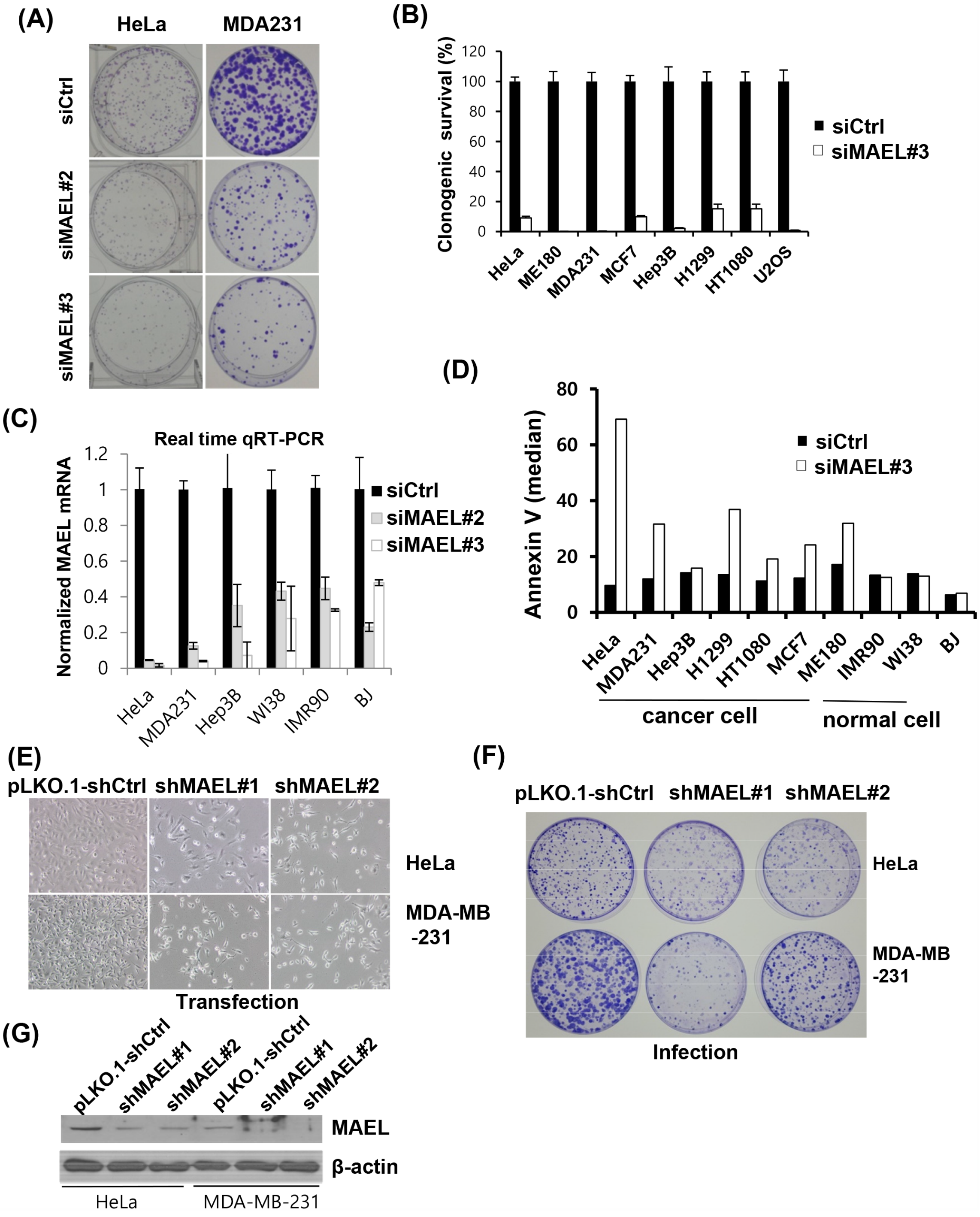

Supplementary Figure S1: MAEL is a cancer cell specific survival factor. A, B. Clonogenic survival assays were used to compare the effects of MAEL depletion on survival of various cancer cell lines with that of HeLa cells. Representative dishes of transfected cells (A) and Plot of clonogenic survival percentage (B). **C.** Real time PCR quantification of MAEL in cancer cell lines and normal cells transfected with siRNA. **D.** Apoptosis evoked by MAEL depletion was measured based on the median values of the fluorescence intensity of annexin V-stained cancer cells and normal cells transfected with siRNA. **E.** Light microscope image of HeLa cells and MDA231 cells transfected with pLKO-shMAEL#1, #2 for 72 hours (x100 magnification). **F.** Clonogenic cell survival of HeLa and MDA-MB-231 cells infected with shMAEL encoding lentivirus. **G.** Western blotting confirming the knockdown efficiency of shMAEL encoding plasmids.

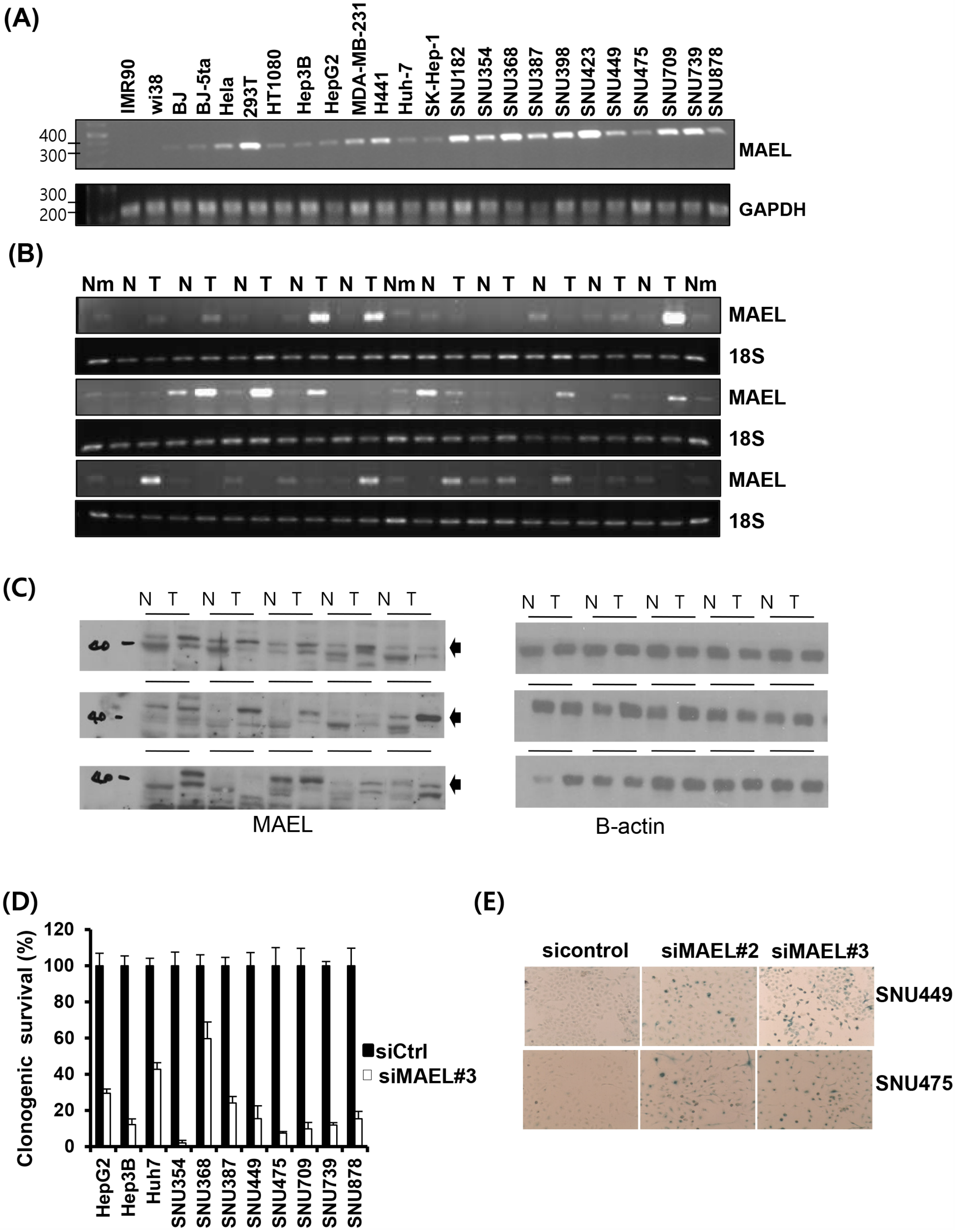

Supplementary Figure S2: MAEL isoform 3 is overexpressed in cancer cell lines and tumor tissues from HCC patients. **A.** The mRNA expression of MAEL in various cancer cell lines including HCC and normal cells. **B, C.** The MAEL expression in paired HCC samples (T) and adjacent non-tumor liver tissues (N) was determined using RT-PCR (B) and Western blotting (C). 18S rRNA and β-actin were used as loading controls. **D, E.** The effect of MAEL depletion on cell survival (D) and senescence (E) was confirmed in various HCC cell lines.

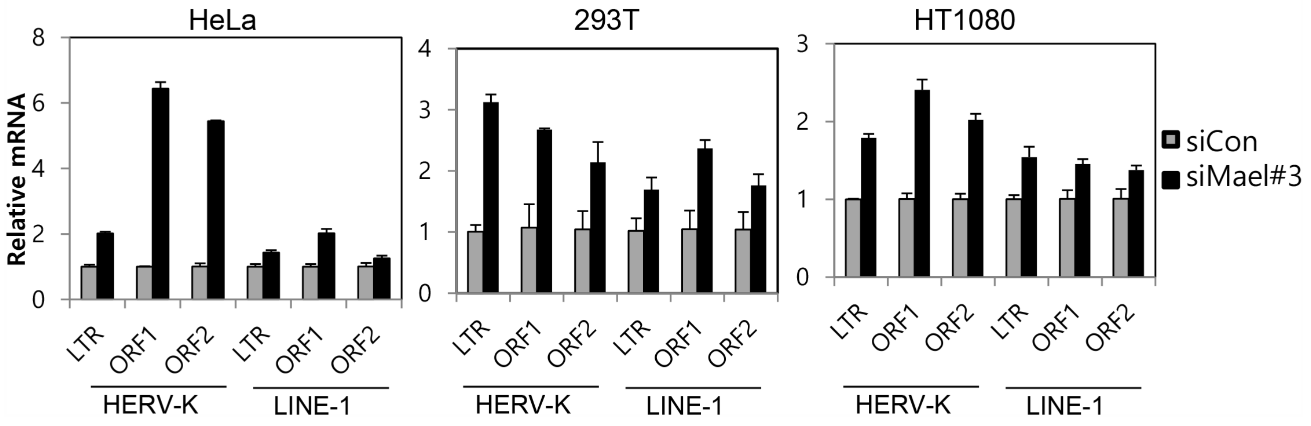

Supplementary Figure S3: MAEL depletion increases the mRNA of retrotransposon. HERV-K, LINE-1 mRNA in MAEL depleted cancer cell lines, HeLa, 293T, HT1080 cells was quantified with Real time PCR. The primers detecting LTR, ORF1 and ORF2 in each transposon were used for analysis.
